# Risk behavior in patients with severe mental disorders: a prospective study of 121,830 patients managed in rural households of western China

**DOI:** 10.1186/s12888-018-1709-8

**Published:** 2018-05-18

**Authors:** Yuanyuan Liu, Xiang Liu, Hong Wen, Dan Wang, Xianmei Yang, Weiwei Tang, Yaxi Li, Tao Zhang, Min Yang

**Affiliations:** 1 Department of Epidemiology and Health Statistics, West China School of Public Health, Sichuan University, Chengdu, Sichuan People’s Republic of China; 2 Department of Health and Social Behavior, West China School of Public Health, Sichuan University, Chengdu, Sichuan People’s Republic of China; 3 Sichuan Provincial Mental Health Centre, Mianyang, Sichuan People’s Republic of China; 4 West China Research Center for Rural Health Development, Sichuan University, No.17 Section 3, South Renmin Road, Chengdu, Sichuan 610041 People’s Republic of China

**Keywords:** Risk behavior, Severe mental disorders, Prospective study, Rural households, China

## Abstract

**Background:**

The management of severe mental disorder (SMD) patients in communities is an important initiative of healthcare reform in China. Yet the effects in terms of risk behavior of patients are unclear, particularly in rural areas. This study aims to examine high risk behaviors, changing trends, and possible associated factors among SMD patients in the rural areas of western China.

**Methods:**

This analysis examined incidence rate (IR) of high risk behavior of 121,830 managed SMD patients in rural area of Sichuan province, based on data from the national system from 2006 to 2013. Register rate, high risk behavior IR, and time distribution and area distribution of high risk behavior were described. Two-level Poisson regression model was used to analyze associates of high risk behavior of patients, which include demographic characteristics at individual level, socio-economic condition and health system indicators at region level.

**Results:**

It was revealed that 6804 (5.58%) of managed patients were involved in 17,220 high risk behavior events, which gave an overall IR of 0.0998 (per person year) on the basis of 172,564 person years of follow-up. The IR varied widely across municipalities, in the range of 0.0305–0.3397. The IR of high risk behavior in the cohort had increased since 2006, and peaked by 2011, at 0.2392. At the individual level, males aged 25 to 44, who were unmarried and in poverty, illiterate or semiliterate, had a family history of mental disorders and antipsychotic treatments, longer duration illnesses, were associated with an increased IR risk. At the regional level, higher psychiatric practitioner visits and the lower annual net income of rural residents per capita, were associated with an increased IR risk.

**Conclusions:**

This is the first large prospective study that revealed the current situation of the register rate, high risk behavior incidence rate in SMD patients in rural area of western China, and examined associates and the differences of high risk behavior of patients among municipalities. The findings may provide evidences that lead to guide prevention and control for risk behavior in SMD patients in rural areas of China, as well as to improve mental health services for this population. It could provide some reference for other developing countries too.

## Background

In China, severe mental disorders (SMD) include six types: schizophrenia, schizoaffective disorder, delusional disorder, bipolar disorder, psychotic disorder due to epilepsy, and mental retardation with mental disorders [1]. It is estimated that there are about 16 million individuals with SMD in China [2], which translates to serious public health and societal problems associated with high disease burdens [3]. In order to reduce these immense social and economic burdens, as well as to protect the legal rights of patients with serious mental disorders, the Chinese government has undertaken many efforts over the last decade, as part of the reform of China’s mental health system [4, 5]. In 2005, the National Continuing Management and Intervention Program for Psychoses (also known as Central Government Support for the Local Management and Treatment of Severe Mental Illnesses Project or “686 Project”) [6, 7] was launched to rebuild China’s public health infrastructure following the SARS epidemic. The “686 Project” was established using the WHO’s recommended strategy for integrating hospital- and community-based mental health services. Following a decade long trial in selected regions, the “686 Project” moved beyond the initial pilot phase toward a process of scaling up community mental health services throughout the country [8]. In 2009, the management of psychoses was included in the National Basic Public Health Services. A guideline of China’s National Basic Public Health Services (2011) specified services for the management of psychoses [9]. In 2012, the Ministry of Health (now the National Health and Family Planning Commission, NHFPC) officially issued a government document: Chinese Managerial and Treatment Regulations for Severely Mentally Disordered Patients (also known as the Working Criteria on Management of Psychoses) [1]. Subsequently, China’s initial national mental health legislation was adopted by the Standing Committee of the National People’s Congress, with the law taking effect in 2013 [10, 11]. The aim of the legislation was to promote mental health, improve health service quality, as well as protect patients with serious mental disorders. One of the most important targets in the management of patients with SMD was to prevent and reduce violent behavior that caused harm to themselves or others [12].

Despite significant political progress with quite comprehensive and instructive nationwide policies in place, the development of mental health service systems and service delivery in China still faces many problems. There exist wide disparities among provinces and regions in terms of socio-economic and developmental levels. In several underdeveloped western areas, the mental health service reform process has been slowed by the poor understanding of public health policies, lack of human health resources, and the required professional skills [4]. Sichuan province possesses the largest population in western China. Its population reached 91.33 million in 2013, with 65 million in the rural areas [13]. It is estimated that there were more than 800,000 patients with SMD in Sichuan province in 2013 [14]. Yet research into the management effects of the system, in terms of risk behaviors in patients with SMD in western China, particularly in rural areas [15],^1^ has rarely been reported [16, 17]. There has been little data available with which to assess the quality of the management of psychoses services locally, or on a large scale, or the efficiency of government initiatives for mental health reform in China [18, 19].

To fill the gap, this study aimed to gather evidence to address the following questions: What is the current situation of risk behavior in patients with SMD and received community services in the rural areas of Sichuan province, and are there any areas with differences in risk behaviors? Are there any changes in the trends of risk behavior outcomes of managed patients in recent years? What are the associated factors with the risk behavior of patients? Might regional socioeconomic factors or health system factors be impacting the risk behavior outcomes of patients? The answers to the above questions will likely assist with the enhancement of prevention and control for risk behavior in patients with SMD in the rural areas of western China. Further, it will help to improve mental health care and services for this population through associated health agencies.

## Methods

### Data sources

The dataset for this study was obtained from the National System of Basic Information Collection and Analysis for Psychoses (abbreviated as **NS**). The NS initiated a pilot trial in 2006, which was scaled up to practically the entire country by 2010. The data system was developed by the NHFPC [1], and managed by professionals at the Centers for Disease Control and Prevention (CDCs) and the Mental Health Centers (MHCs) of China. Primarily, the data system employed two approaches to engage patients. Initially, they identified patients who were referred from psychiatric hospitals or related departments in general hospitals. Secondly, they screened patients for possible psychosis who were referred from the China Disabled Persons’ Federation (CDPF), local communities, township health centers, neighborhoods, or village committees. These patients were subsequently examined by psychiatrists, and those who met the diagnostic criteria (ICD-10 or DSM-IV) [20] for psychotic disorders were evaluated for their disease condition by the national working group. This evaluation was focused on a risk assessment of violence, mental symptoms, self consciousness, social functionality, adverse drug reactions, and other serious physiological diseases [4]. The frequency of evaluation in a follow-up was determined by patient’s disease condition. Patients whose diseases were stable, or basically stable, were followed at least once every three months. The criteria for stable or basically stable were as follows: low risk score at level 0-2 (see the definitions of risk levels in the next section), no significant mental symptoms, no adverse drug reactions or serious physiological diseases, good self consciousness, and social functionality. Otherwise, patients were classified as being in an unstable condition, who received weekly or monthly follow-ups [1]. Sichuan was one of the first provinces to establish NS in China, which commenced in May 2006. Up to December 2013, 155,478 patients were registered in the provincial system. In the whole follow-up period, patients may enter or leave the cohort at any time. Their follow-up period reached 7.35 years at the longest, with a mean follow uptime of 1.40 years (SD: 0.87). Following data integrity checks, we obtained 144,243 valid cases, with the cases having error or missing values in outcome variables excluded. Among them 121,830 (84.5%) patients lived and were managed in rural areas, whereas the remaining 22,413 (15.5%) patients resided in urban areas.

### Study measures

We considered the risk behavior of patients as a key measure for the quality of the management of psychoses services. Risk behavior was defined on a scale of from 0 to 5. Level 0 (L0) did not refer to any of the following behaviors indicated in levels 1 to 5. Level 1 (L1) risk behavior included verbal threats and shouts to others, but no physically destructive behavior. Level 2 (L2) included the destruction of property, but was home limited, and with persuasion could be stopped. Level 3 (L3) obviously included the destruction of property regardless of the occasion, which with attempted persuasion could not be stopped. Level 4 (L4) was defined by continuous destructive behavior to individuals or property regardless of the occasion, which with attempted persuasion could not be stopped. Level 5(L5) was defined by any violent behavior against individuals using controlled dangerous weapons, or other dangerous behavior such as arson, blasts, etc., regardless of the occasion. According to Chinese Managerial and Treatment Regulations for Severely Mentally Disordered Patients, the risk level between L0 and L2 was classified as “low risk behavior” and L3 – L5 as “high risk behavior” [1]. In this study, the number of high risk behavior incidents of each patient that were recorded in the follow-up period in the dataset, were defined as outcome variables. The follow-up year of each patient was employed as a denominator to define the incidence of high risk behavior.

To examine potential associated factors with the incidence of risk behavior of patients, we considered several groups of variables. The first group pertained to demographic characteristics at the individual level, which were released by the NS, such as gender, age, family history of mental disorder, marital status, ethnic group, education qualification, economic status, type of diagnosis, duration of illness, antipsychotic treatments, and participation in the “686 Project”. The second group of variables involved socioeconomic conditions at the region or municipal level, such as the **a**nnual **n**et **i**ncome of rural residents **p**er **c**apita (ANIPC), **e**mployment **p**roportion of rural residents (EP%), and **p**er **c**apita **m**inimal **l**ivelihood **f**unds for rural residents under basic provision protection (PCMLF). The above regional variables were collected from the Sichuan Statistical Yearbook (2014), which are the official statistics of 2013 [13]. The third group of variables was related to health system indicators that were collected by the Mental Health Center of Sichuan Province in 2013. These indicators were specific to mental health service for patients with SMD at the regional level. Variables included the **n**umber of **a**gencies for **s**evere **m**ental **d**isordered (NASMD), **n**umber of inpatient **b**eds for **s**evere **m**ental **d**isordered (NBSMD), **n**umber of **p**sychiatric **p**ractitioners (including assistant psychiatric practitioners) per one hundred thousand people (NPP/100,000), and the **a**verage number of **f**ollow-ups for **p**atients in the rural region (AFP).

### Statistical analyses

Since the incidence of high risk behavior was infrequent, and hierarchical structure in this dataset is presented with individuals nested within municipalities, multilevel Poisson regression was used to model incident rate ratios (IRRs), and to examine associated factors. At the individual level, demographic characteristic variables assisted with the identification of subgroups for high risk screening and prevention. At the regional level, municipality socioeconomic variables helped to assess the extent to which contextual factors might impact individual behaviors in addition to individual risk factors. Similarly, health system indicators at the municipal level were utilized to examine how health system performance might impact individual risk behaviors, with the aim being that evidence might shed light on the best use of health resources.

Three two-level Poisson models with random intercepts were constructed to examine risk factors at different levels and by variable groups. Model 0 had no variables, but was used to test regional clustering effects and estimate regional variations in high risk behavior incidence as a benchmark. Model 1 included individual-level factors, whereas Model 2 incorporated both individual-level and region-level variables. Model fits, following the addition of each group of variables, was evaluated by pseudo-AIC criteria [21]. The total years of follow-up during the study period, was the offset variable (underlying person-time) for the regression models. The General Ward *χ*^2^ statistic was employed to test significance of random coefficients.

These studies received Institutional Review Board approval for research involving human subjects from Sichuan University and Mental Health Center of Sichuan Province, where the research was conducted. The analysis was conducted using SAS 9.3 and SPSS 19.0.

## Results

**Fig. 1:**
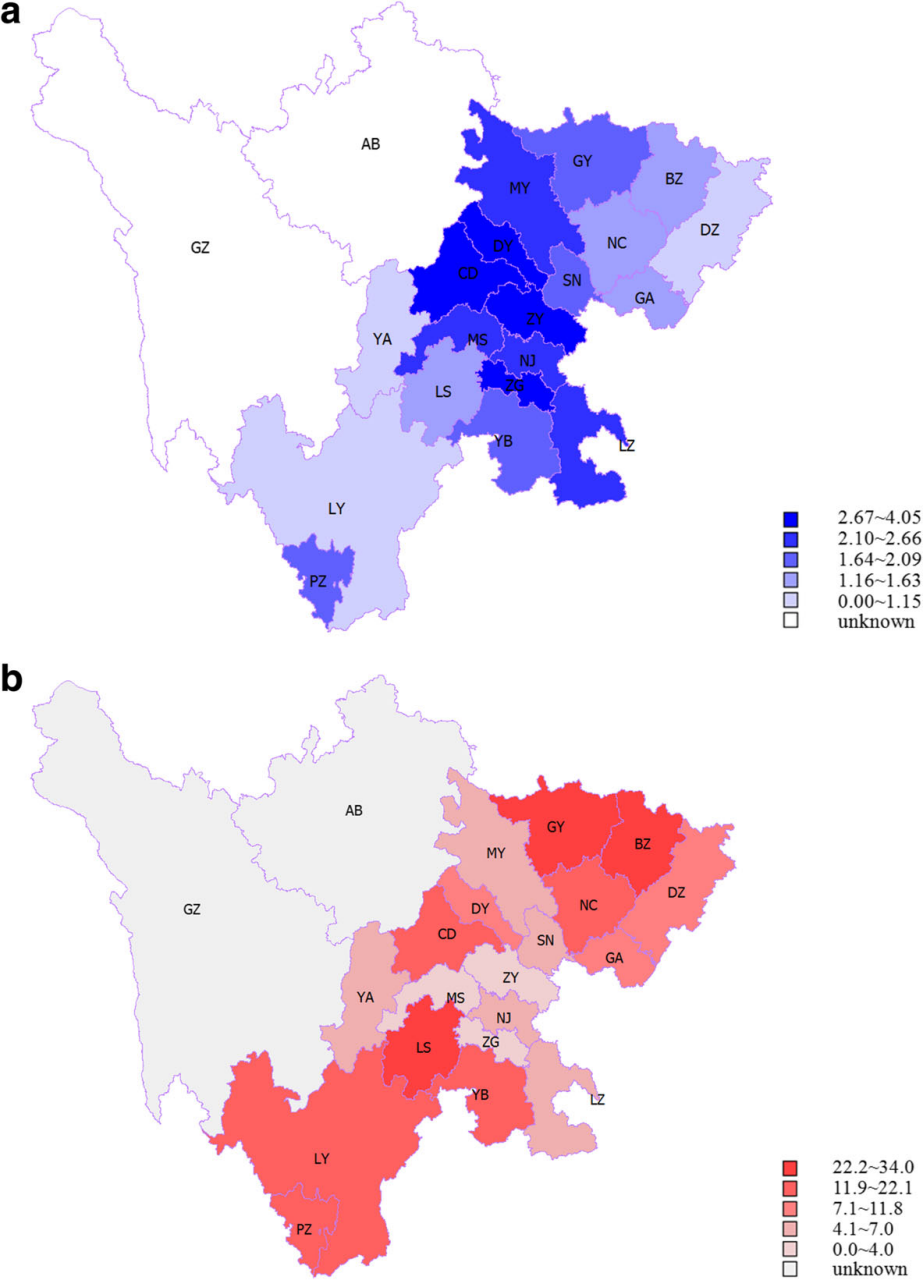
**a** Distribution of registration rate (‰) of patients with SMD in rural areas in Sichuan province (2006 – 2013). **b** Distribution of incidence rate (%) of high risk behavior in patients with SMD in rural areas in Sichuan (2006 – 2013)

Registered patients were distributed in 19 out of 21 municipalities in Sichuan province, as shown in Fig. 1a. The missing cases in two municipalities were due to the fact that there were no recognized mental health care agencies in the regions, and the NS system was not mature enough to generate reliable management data at the time when the study data were collected. As these two municipalities accounted for 2.4% of the total population in the province, the analysis in the study represented 97.6% of the population in Sichuan.

The overall registration rate of patients was 1.92 per thousand population, in the range of from 0.54 to 4.05 ‰ across municipalities (see Table 1). Over 8 years, 6804 (5.58%) out of 121,830 managed patients were involved with 17,220 instances of high risk behavior, which gave an overall incidence rate 0.0998 (per person-year) on the basis of 172,564 person years of follow-up. As shown in Table 1 and Fig. 1b, the incidence rate varied significantly across municipalities, in the range of from 0.0305 to 0.3397 (per person-year).

**Table 1:** Regional distribution of patients with SMD and incidence of high risk behaviors of patients in rural households of Sichuan province in cohort, from 2006 to 2013^a^

Regions	Registered patients			High risk behavior			
	Population (million)	N_1_	Registration rate (‰)	N_2_	No. of high risk behaviors	Follow-up person years	Incidence rate (%)
1 CD	4.59	18,588	4.05	752	3593	30,118.68	11.93
2 ZG	2.17	8039	3.70	271	508	12,620.50	4.03
3 PZ	0.52	853	1.64	83	138	622.63	22.16
4 LZ	3.56	7490	2.10	355	707	10,013.31	7.06
5 DY	2.75	7883	2.87	617	1225	12,933.39	9.47
6 MY	3.89	8916	2.29	430	901	15,157.51	5.94
7 GY	2.38	3897	1.64	447	1307	4971.04	26.29
8 SN	2.82	4645	1.65	159	279	6745.85	4.14
9 NJ	3.30	7745	2.35	303	652	13,035.34	5.00
10 LS	2.38	3637	1.53	384	994	4217.26	23.57
11 NC	5.82	7174	1.23	536	1362	7429.37	18.33
12 MS	2.53	5651	2.23	119	257	7523.61	3.42
13 YB	4.44	8093	1.82	631	1244	9646.55	12.90
14 GA	3.80	4392	1.16	292	424	5404.53	7.85
15 DZ	5.48	5630	1.03	221	667	7088.68	9.41
16 YA	1.14	1169	1.03	63	85	1382.37	6.15
17 BZ	3.12	4463	1.43	720	2167	6378.79	33.97
18 ZY	4.18	11,143	2.67	289	485	15,888.46	3.05
19 LY	4.46	2422	0.54	132	225	1386.21	16.23
Total	63.35	121,830	1.92	6804	17,220	172,564.09	9.98

^a^Population refers to agricultural population in 2013

*N*_1_: the number of registered patients with SMD

*Register rate*: the rate of registered patients with SMD in local population. Limited by variable objective conditions, registration rate is lower than the prevalence rate of SMD. *Register rate* = *N*_1_/ Population

*N*_2_: the number of high risk behavior patients in registered patients with SMD. As mentioned in 2.1.1, whether a patient is classified into a high risk group is according to the highest risk level in their follow-up period. If they were once, or more times, evaluated as L3 to L5, they were classified into a high risk group

*No. of high risk behaviors*: the number of occurrences for high risk behavior in registered patients with SMD. In this study, there were 115,026 patients whose *no. of high risk behaviors* was 0, and the remaining patients with *no. of high risk behaviors* ranged from 1 to 40

*Follow-up person years*: the sum of the years of follow-up time of registered patients with SMD

*Incidence rate*: the incidence rate of high risk behavior in registered patients, and the follow-ups in person years were used as the denominator to define the incidence of high risk behavior. *Incidence rate* = *No of high risk behaviors* / *Follow-up person years*

To avoid the values being too small, all incidence rates in Tables 1, 2, 3 and 4 were multiply by 100%

Examining the registration number by year, the results in Table 2 suggest that the NS system was in a pilot period spanning 2006 – 2009, and only a small proportion of patients were managed in the system. The rapid increase in the number of registered patients following 2009 suggested the positive impact of national policy and investment in the system since 2009. The quality of the data was, at least, improved in terms of coverage and completion.

**Table 2:** Time distribution of high risk behavior of patients with SMDs in rural households of Sichuan province

Year	N_1_	N_2_	No. of high risk behaviors	Follow-up person years	Incidence rate (%)
2006	6	0	0	1.27	0.00
2007	40	2	2	20.87	9.58
2008	75	1	1	57.81	1.73
2009	320	16	16	150.99	10.60
2010	6046	254	371	2557.09	14.51
2011	66,549	3852	6045	25,271.64	23.92
2012	97,668	2689	6141	75,017.99	8.19
2013	101,581	1930	4644	69,321.44	6.70

The temporal trend in the incidence rate of high risk behavior (Table 2), was observed to increase and peak at 0.2392 (per person year) by 2011, followed by a downward trend. This might have been a reflection of the management effects following the development of community mental health services in recent years. The violent behavior of patients comprised one of the management and preventive targets of the services.

A further examination of changing trends in the high risk behavior incidence rate by municipality, from 2011 to 2013 (Table 3), revealed that except for one municipality, an annual downward trend of from between 5 and 77% in the outcome was observed across all municipalities, with an overall annual rate of decrease of 47%.

**Table 3:** Incidence rate of high risk behavior by region of rural Sichuan province from 2011 to 2013^a^

Region	2011			2012			2013			
	No. of high risk behaviors	Follow-up person years	Incidence rate (%)	No. of high risk behaviors	Follow-up person years	Incidence rate (%)	No. of high risk behaviors	Follow-up person years	Incidence rate (%)	Annual rate of change^a^
1 CD	1109	4031.33	27.51	1418	12,234.69	11.59	1061	13,743.52	7.72	−0.47
2 ZG	254	1691.39	15.02	160	5333.33	3.00	78	5492.96	1.42	−0.69
3 PZ	4	41.07	9.74	11	168.20	6.54	123	409.32	30.05	0.77
4 LZ	337	1280.08	26.33	235	4234.23	5.55	88	4378.11	2.01	−0.72
5 DY	751	2722.03	27.59	115	5066.08	2.27	272	4857.14	5.60	−0.55
6 MY	448	2370.25	18.90	334	6972.86	4.79	81	5625.00	1.44	−0.72
7 GY	468	807.15	57.98	590	2327.42	25.35	244	1788.86	13.64	−0.51
8 SN	123	1470.52	8.36	77	2851.85	2.70	27	1985.29	1.36	−0.60
9 NJ	337	2523.60	13.35	260	5816.55	4.47	44	4313.73	1.02	−0.72
10 LS	244	422.82	57.71	423	1696.07	24.94	327	2070.93	15.79	−0.48
11 NC	572	1460.63	39.16	505	3454.17	14.62	259	2273.92	11.39	−0.46
12 MS	88	1145.18	7.68	92	3076.92	2.99	57	3097.83	1.84	−0.51
13 YB	309	950.19	32.52	655	4428.67	14.79	269	4151.23	6.48	−0.55
14 GA	247	784.79	31.47	85	2537.31	3.35	71	1977.72	3.59	−0.66
15 DZ	172	710.48	24.21	287	3235.63	8.87	205	3005.87	6.82	−0.47
16 YA	52	137.49	37.82	18	640.57	2.81	15	595.24	2.52	−0.74
17 BZ	297	526.01	56.46	496	3167.31	15.66	1369	2669.14	51.29	−0.05
18 ZY	222	1977.65	11.23	183	7093.02	2.58	38	6440.68	0.59	−0.77
19 LY	11	218.03	5.04	197	693.91	28.39	16	465.12	3.44	−0.17
Total	6045	25,270.70	23.92	6141	74,981.68	8.19	4644	69,313.43	6.70	−0.47

^a^Annual rate of change==$\sqrt[n]{a_n/{a}_0}$-1

According to the descriptive analysis results of Table 4, it may be seen that patients with following characteristics were more likely to have high risk behaviors: male, aged 15 to 44, national minority, a family history of mental disorders, unmarried, living in poverty, illiterate/semiliterate, illness duration of 10 to 29 years, no antipsychotic treatment, not participating in “686 Project”, and follow-up time of less than 4 years.

**Table 4:** Distribution of high risk behavior by characteristics of patients with SMDs in rural households of Sichuan province

Characteristic	N_1_	N_2_	No of high risk behaviors	Follow-up person years	Incidence rate (%)
Gender					
1 = male	59,957	3737	9707	84,164.46	11.53
2 = female	61,873	3067	7513	88,399.63	8.50
Age					
1 = 15 to 24	5932	275	783	7424.24	10.55
2 = 25 to 44	45,022	2859	7232	63,412.73	11.40
3 = 45 to 59	37,632	1952	4969	54,161.07	9.17
4 = ≧60	33,244	1718	4236	47,566.04	8.91
Nationality					
1 = Han	120,428	6739	17,113	171,753.73	9.96
2 = Minority	1402	65	107	810.36	13.20
Family history of mental illness					
0 = no	116,988	6472	16,376	165,982.10	9.87
1 = yes	4842	332	844	6581.99	12.82
Marital status					
1 = married	73,619	3679	8988	105,017.25	8.56
2 = unmarried	46,396	3033	8039	64,914.40	12.38
3 = NK	1815	92	193	2632.44	7.33
Economic status					
1 = poverty^a^	78,932	4867	12,883	113,025.02	11.40
2 = non-poverty	17,134	694	1744	25,198.74	6.92
3 = NK	25,764	1243	2593	34,340.33	7.55
Education degree					
1 = illiteracy/semiliterate	42,697	2404	6309	59,656.04	10.58
2 = elementary education and above	74,325	4130	10,374	106,046.29	9.78
3 = NK	4808	270	537	6861.77	7.83
Diagnosis type					
1 = schizophrenia	103,412	6055	15,362	149,703.01	10.26
2 = schizoaffective disorder	1849	121	256	2143.59	11.94
3 = delusional disorder	480	20	72	673.90	10.68
4 = bipolar disorder	2995	168	415	3918.84	10.59
5 = psychotic disorder due to epilepsy	4980	234	693	6561.95	10.56
6 = mental retardation with mental disorders	8114	206	422	9562.81	4.41
Duration of illness					
1 = < 10 years	46,009	2305	5544	58,635.88	9.45
2 = 10 to 19 years	36,468	2212	5689	54,715.99	10.40
3 = 20 to 29 years	22,381	1333	3533	34,039.29	10.38
4 = ≧30 years	16,972	954	2454	25,172.93	9.75
Antipsychotic treatment					
0 = no	27,489	1549	4193	35,999.18	11.65
1 = yes	94,341	5255	13,027	136,564.91	9.54
Participation in 686 project					
0 = no	14,522	1300	2613	22,780.70	11.47
1 = yes	107,285	5504	14,607	149,752.54	9.75
3 = NK	23	0	0	30.84	0.00
Follow-up time					
1 = < 2 years	84,477	3978	8787	83,345.00	10.54
2 = 2 to 4 years	37,196	2817	8401	88,440.56	9.50
3 = >4 years	157	9	32	778.53	4.11

^a^Poverty refers to the economic status that is below the local poverty line by income level

As the univariate analysis did not take into consideration of the effects of other variables, multivariate analyses were further carried out to control the potential confounders. A baseline Model 0 that did not include any variables estimated the variance of random effects of the outcome at municipal level as 0.486 (*χ*^2^ = 8.922, *P* = 0.003). This implied that the incidence rates of the high risk behavior were significantly varied among municipalities. Having considered individual level factors, Model 1 in Table 5 shows that several of these factors were significantly associated with high IRR, rather than their reference group: aged between 25 and 44, unmarried, having family history of mental disorders, and on antipsychotic treatment; longer duration of illness increases risk, with an IRR range of from1.125 (10 to 19 years), 1.153 (20 to 29 years), to 1.165 (≧30 years), compared to an illness duration of < 10 years.

**Table 5:** Two-level Poisson regression analyses for associates with high risk behavior of patients with severe mental illness in rural households of Sichuan Province

Variables	Model1		Model2	
Fixed effect	Coefficient	IRR(95% CI)	Coefficient	IRR(95% CI)
Regional Level				
NPP (per 100,000 pop.)	–	–	0.494*	1.638(1.001, 2.682)
ANIPC(RMB Yuan)	–	–	− 8.039*	0.000(0.000, 0.558)
EP(%)	–	–	− 2.055	0.128(0.000, 35.929)
PCMLF(RMB Yuan)	–	–	−0.001	0.999(0.998, 1.000)
AFP	–	–	0.100	1.105(0.948, 1.288)
Individual Level:				
Gender				
1 = male	–	–	–	–
2 = female	− 0.205**	0.814(0.787, 0.842)	− 0.205**	0.814(0.787, 0.842)
Age				
1 = 15 to 24	–	–	–	–
2 = 25 to 44	0.128**	1.137(1.053, 1.227)	0.128**	1.137(1.054, 1.227)
3 = 45 to 59	−0.068	0.935(0.862, 1.013)	−0.068	0.935(0.863, 1.013)
4 = ≧60	−0.125**	0.882(0.812, 0.958)	−0.125**	0.882(0.812, 0.959)
Nationality				
1 = Han	–	–	–	–
2 = Minority	−0.176	0.839(0.685, 1.028)	−0.175	0.839(0.685, 1.028)
Family history of mental illness				
0 = no	–	–	–	–
1 = yes	0.227**	1.254(1.170, 1.344)	0.227**	1.254(1.170, 1.345)
Marital status				
1 = married	–	–	–	–
2 = unmarried	0.223**	1.250(1.206, 1.295)	0.223**	1.250(1.206, 1.295)
Economic status				
1 = poverty	–	–	–	–
2 = non-poverty	−0.494**	0.610(0.579, 0.642)	−0.494**	0.610(0.579, 0.643)
Education level				
1 = illiteracy/semiliterate	–	–	–	–
2 = elementary education and above	−0.123**	0.884(0.855, 0.914)	−0.123**	0.884(0.855, 0.914)
Diagnosis type				
1 = schizophrenia	–	–	–	–
2 = schizoaffective disorder	−0.044	0.957(0.845, 1.083)	−0.045	0.956(0.845, 1.082)
3 = paranoid psychosis	0.042	1.043(0.826, 1.316)	0.041	1.042(0.826, 1.315)
4 = bipolar affective disorder	−0.123*	0.885(0.802, 0.976)	−0.123*	0.884(0.802, 0.975)
5 = mental disorders due to epilepsy	−0.262**	0.769(0.712, 0.831)	−0.262**	0.769(0.712, 0.831)
6 = mental retardation with mental disorders	−1.096**	0.334(0.302, 0.370)	−1.096**	0.334(0.302, 0.370)
Duration of illness				
1 = < 10 years	–	–	–	–
2 = 10 to 19 years	0.118**	1.125(1.084, 1.168)	0.118**	1.125(1.084, 1.168)
3 = 20 to 29 years	0.142**	1.153(1.104, 1.204)	0.142**	1.153(1.104, 1.204)
4 = ≧30 years	0.152**	1.165(1.106, 1.226)	0.152**	1.165(1.107, 1.226)
Antipsychotic treatment				
0 = no	–	–	–	–
1 = yes	0.039*	1.040(1.002, 1.079)	0.039*	1.040(1.003, 1.079)
Participation in 686 project				
0 = no	–	–	–	–
1 = yes	−0.266**	0.767(0.734, 0.801)	− 0.266**	0.766(0.733, 0.801)
Random effect				
Level 2: Region $\left({\sigma}_{u_0}^2\right)$	0.502(0.168)**		0.417(0.165)*	

*P<0.05, **P<0.01

Full description of regional variables as follows: **a**nnual **n**et **i**ncome of rural residents **p**er **c**apita (**ANIPC**), **e**mployment **p**roportion of rural residents (**EP%**), and **p**er **c**apita **m**inimal **l**ivelihood **f**unds for rural residents under basic provision protection (**PCMLF**), **n**umber of **p**sychiatric **p**ractitioners (including assistant psychiatric practitioners) per one hundred thousand people (**NPP/100,000**), the **a**verage number of **f**ollow-ups of **p**atients in rural region (**AFP**)

Factors that seemly had lower IRR than their comparison groups were: female gender, age ≧60, not in poverty, with elementary education and above; bipolar affective disorder, mental disorders due to epilepsy and mental retardation, with mental disorders having a lower IRR compared to schizophrenia. Patients participating in the “686 Project” appeared to have lower risk (IRR = 0.767) than otherwise. Model 2 incorporated municipal factors into Model 1, which reduced the municipal level variance for random effects, from 0.502 (*P* = 0.003) in Model 1, down to 0.417(*P* = 0.011). Higher numbers of psychiatric practitioners (including assistant psychiatric practitioners) per 100,000 population (NPP/100,000), and the lower annual net income of rural residents per capita (ANIPC), were associated with an increased IRR. They accounted for only about 17% of the difference in the incidence rate of high risk behavior of patients among municipalities. With adjustment for municipality factors, the effects of all individual factors on the incidence rate remained unchanged as in Model 1.

## Discussion

The NS is the only mental health themed surveillance system in China [17, 18]. This is the first large prospective study based on the NS of Sichuan province to reveal the current situation of the registration rate, high risk behavior incidence rate in patients with SMD in the rural areas of western China. Individual and regional factors were examined in association with the high risk behavior incidence rates of patients, and the differences of the high risk behavior of patients among municipalities in Sichuan province. These findings may provide useful evidence to guide the prevention and control of risk behaviors in patients with SMD in the rural areas of western China, as well as to improve mental health services for this population.

### The registration rate of patients with SMD

A previous large epidemiological survey, spanning 2001 – 2005 in four provinces of China reported an adjusted 1-month prevalence of psychotic disorder at 1.0% (95% CI: 0.8-1.1) [2]. A meta analysis of 2,284,957 Chinese individuals reported a lifetime prevalence of schizophrenia alone as 0.36% in 1990, which increased to 0.83% in 2010 [22]. The Chinese government set the goal for the national detection rate of severe mentally disordered patients for the NS system at 0.35% in 2014 [23].

The registered patients of rural areas with SMD in the NS system in this study was at 0.192%, which was lower than the prevalence rate of patients with SMD in the general population, and lower than the national detection rate target. This was because approximately two thirds of the identified patients were not included in the NS system for community services. They were: (1) patients who were completely cured, and (2) patients who could not be cured; however, at a degenerating stage were harmless to society. This means that the NS system cannot be used to report the true prevalence of SMD in the population. However, from 2006 to 2013, the registration rate gradually rose from almost null to 0.192%, which was particularly the case after the Chinese government launched the Phase 1 NS system at the national scale in 2011. This might reflect a positive effect of system efficiency for the management of more patients in rural areas.

The large variation in the registration rates, from 0.405% in CD to 0.054% in LY, may reflect the quality of the NS system among municipalities related to multiple regional contextual factors such as socioeconomics, access to health services, health workforce resources, and education. As the capital of Sichuan province, CD had the highest per capita annual net income of rural residents in the province in 2014 [13]; hence, it benefited from 27 mental health agencies, 0.46‰ per capita inpatient psychiatric beds, and 0.037‰ per capita psychiatric practitioners (including assistant psychiatric practitioners). In contrast, the LY Autonomous Prefecture is located in a mountainous area, with the per capita annual net income of rural residents ranked at the bottom third of the province. It has only three mental health agencies, 0.17‰ per capita inpatient psychiatric beds, and 0.007‰ per capita psychiatric practitioners (including assistant psychiatric practitioners). Therefore it is critical to examine, as in this study, how disparities in these municipal level factors might impact patient outcomes, such as high risk behavior.

### The incidence rate of high risk behavior by municipality

This study demonstrated that from 2011 to 2013, when the NS system was solidly in place, there was a significant downward trend in the incidence rate among the studied patient population, from 23.92% per person year (2011) to 6.70% (2013). This downward trend reflected a positive effect of the health care management of SMD, particularly the development of community mental health services. More specifically, such declining trends were observed among all municipalities except for one PZ, where the incidence rate increased from 9.74 to 30.05% during the three year period. There are a number of possible reasons to explain the rise of such an outcome in this municipality. First, PZ is a typical region of migrants with a large floating population proportion who are more vulnerable to mental disorders than others [24]. Second, PZ is on the border with the LY Yi Minority Autonomy Prefecture, which has much better mental health care services. Many patients of Yi ethnic minority migrated from LY to work or live in PZ; hence, they were registered as crossing the border. Third, according to an internal report, many new patients came into the system since 2012 for the benefits of the NS, such as free treatment, free medical examination, and free training, etc. All these factors resulted in the unstable incidence rate in PZ. Another notable rise of the high risk behavior incidence was the BZ municipality, where the outcome was 56.46% (2011), 15.66% (2012), and up to 51.29% (2013). A common reason shared with the LY was a large proportion of floating population from BZ to other locations, due to its poor economic conditions, i.e. the lowest per capita net annual income of rural residents in Sichuan province. Many migrants returned to their hometowns after they were found to be mentally ill.

These findings suggest that both central and provincial health authorities should pay special attention, and perhaps invest more in the development of mental health care services to areas where economic development is low, transportation might be difficult, large populations of migrants and health system indicators are poorer than other regions.

### Associates of high risk behavior at individual level

This study identified a number of factors at the individual level in different categories that were positively associated with an increase of high risk behavior. The demographic factors included being of male gender, between the ages of 25 and 44, an ethnic minority, and unmarried. The socioeconomic factors included illiteracy/semi-literacy and poverty. The disease related factors included having a family history of mental disorders, with schizophrenia diagnosis, and longer illness duration. Environmental factors were not part of the government “686 Project”. Most of those factors were supported by previous national and international studies [25–28]. The mechanisms of their association with risk behaviors have been well examined in the fields of psychiatry and forensic psychiatry, and are mostly known. Patients who were involved in the “686 Project” showed lower risk outcomes. This might indicate that the “686 Project” created benefits for the patients, both from the management level and treatment effect. Future research could examine the predictive power of these factors in order to develop a screening tool for high risk groups for management and treatment purposes in the community.

### Associates of high risk behavior of patients at municipal level

We found that a reduced regional socioeconomic advantage, measured by the annual net income of rural residents per capita, and an increased regional mental health human resource, measured by the number of psychiatric practitioners (including assistant psychiatric practitioners) per 100,000 population, were associated with an increased high risk behavior of patients in the study. Together, these two factors explained about 17% of the outcome variance among municipalities in Sichuan.

The association between socioeconomic disadvantage and worsening mental health outcomes might be explained by the supply and needs relationship [29]. However, an explanation for the association of larger mental health workforce with the increased risk behavior of patients is not intuitive. We speculated on the main cause: the recently increased number of mental health workforce improved the management activity in identifying preventive outcomes such as the risk behavior of patients. In China, psychiatrists and licensed psychiatric nurses are accredited by the NHFPC, psychological counsel or accredited by the Ministry of Human Resources and Social Security, and psychotherapists by both Ministries [4]. In 2004, China only had 16,103 licensed psychiatrists and psychiatric registrars (1.24/100,000 population) and 24,793 licensed psychiatric nurses (1.91/100,000 population) [30], in contrast to 4.15 psychiatrists and 12.97 psychiatric nurses per 100,000 population, respectively, for the average global mental health workforce in the same year [31].

By 2013, in Sichuan province, there were 1621 registered psychiatrists, 1757 (assistant) psychiatrists and 3952 psychiatric nurses in psychiatric hospitals, in conjunction with 25,676 practicing physicians, 41,263 practicing (assistant) physicians, and 24,031 registered nurses in primary healthcare institutions for all primary health care services [32]. The management of mental disorders in communities was only one task of the services, and the professional workforce was small. However, for the NS system services, additional locally trained mental health workers could have been recruited to contribute to the national task of identifying and managing patients with SMD in the community. As a consequence, more risk behaviors of patients would be reported. Further investigations into the quality of services in patient treatment, and the prevention of risk behavior, at both individual and health care facility levels will be required on this account.

### Limitations

A number of limitations in the study should be noted. Firstly, the NS was originally a response to the government’s concern regarding social harmony and stability, as part of the “686 Program” [4]. It only focuses on SMD patients in terms of their violent or socially disruptive behaviors [18]. Meanwhile, information sharing between different surveillance systems was constrained by the incompatibility of their independent software and hardware [33]. As such, the variables employed in this study are limited and non-comprehensive. Secondly, the quality of the data is unoptimistic. The main problems include underreporting, missing values, and errors, which were due to the increasing gap between the demand and supply of mental health care, and the social stigma rooted in the moral and political context [5]. The problem could be higher in rural areas, with less training for rural staff, coupled with increased difficulties in accessing widely dispersed rural residents of regions with poor transportation systems [34, 35]. Quality issues may affect the accuracy of outcomes; however, they should have less impact on risk factor analysis, which is based on relative comparisons. Thirdly, as NS system was built from 2006, the patients enrolled in the first few years were very limited. The incidence rates in 2006-2009 could only represent the cohort, while not the local actual level. Fourthly, accessing data from the system is extremely difficult in China. No report on national psychosis surveillance has yet been released, except for a few papers published by local surveillance staff based on data from their regions [36]. Hence, a nationwide comparison of the study outcome could not be made.

### Implications

The present study provides evidence to support the primary goal of the movement for Global Mental Health [37], and the WHO Mental Health Gap Action Program [38], to promote the scaling up of mental health services in low-income and middle-income countries [39]. Sichuan represents provinces in western China where development in economic and health care have lagged behind the rest of China. This puts mental health care issues in the rural areas of Sichuan on par with those of low income countries. As the researches on risk behavior in patients with SMD were mainly carried in the developed countries, our study findings may fill the knowledge gap regarding the quantity and quality of how the system operates, and to what degree patient risk behaviors were controlled for, and what possible associates might be linked with their risk behavior in developing countries. Changing trends in the registration and incidence rates suggested several positive impacts of the system over the last five years. A further in-depth study of the effects of the system might be a way to progress, based on epidemiological facts. Evidence regarding the significant variations in the incidence of the high risk rate between municipalities with known socioeconomic and human resource factors, and with identified municipal low and high patient outcomes, might advocate for the health authority to make decisions based on health resource distribution with clear localized targets. Risk factors at the individual level identified in this study may be used to develop a risk screening tool to earlier identify patients with the highest risk factors for targeted risk management by grass-level mental health workers. The above findings may be generalized to other developing countries in future.

## Conclusions

The registration rate and incidence rate of risk behavior varied widely across municipalities in rural areas of west China. The factors in association of increased high risk behavior include males, aged 25 to 44, unmarried, in poverty, illiterate or semiliterate, having a family history of mental disorders and antipsychotic treatments, longer duration illnesses at the individual level; and higher psychiatric practitioner visits, the lower annual net income of rural residents per capita at the regional level.

## Abbreviations

“686 Project”: The National Continuing Management and Intervention Program for Psychoses (also known as Central Government Support for the Local Management and Treatment of Severe Mental Illnesses Project)
ANIPC: Annual net income of rural residents per capita
CDCs: Centers for Disease Control and Prevention
CDPF: China Disabled Persons’ Federation
EP%: Employment proportion of rural residents
IR: Incidence rate
IRRs: Incident rate ratios
MHCs: Mental Health Centers
NASMD: Number of agencies for severe mental disordered
NBPHS: National Basic Public Health Services
NBSMD: Number of inpatient beds for severe mental disordered
NHFPC: National Health and Family Planning Commission (previously been called the Ministry of Health)
NPP/100,000: Number of psychiatric practitioners (including assistant psychiatric practitioners) per one hundred thousand people
NS: National System of Basic Information Collection and Analysis for Psychoses
PCMLF: Per capita minimal livelihood funds for rural residents under basic provision protection
SMD: Severe mental disorders

## Footnotes

1 Based to the Household Registration System in China, residents are divided into urban and rural according to their registration in the system no matter where they live. In the aspect of geographical distribution, people natively live in level regions below township level are usually rural household registration [15].
